# Construction of automatic error detection model for English-Chinese translation of cultural heritage terminology based on BERT-BiLSTM-CRF

**DOI:** 10.3389/frai.2026.1894591

**Published:** 2026-08-26

**Authors:** Yesheng Yu, Xubo Liu, Luobin Jin

**Affiliations:** 1 Faculty for College English, Zhejiang Yuexiu University, Shaoxing, Zhejiang, China; 2 Eaton (China) Investments Co., Ltd., Shanghai, China; 3 School of Languages and Cultures, Shaoxing Institute of Technology, Shaoxing, Zhejiang, China

**Keywords:** BERT-BiLSTM-CRF model, cross-lingual semantic modeling, cultural heritage translation, sequence labeling model, translation error detection

## Abstract

For a long time, there has been a lack of effective cultural semantic conversion and automatic detection methods for term inconsistency in the English Chinese translation of cultural heritage terminology. This paper constructs an automatic error detection model for cultural heritage terminology translation based on Bidirectional Encoder Representations from Transformers (BERT), Bidirectional Long Short Term Memory (BiLSTM), and Conditional Random Field (CRF). This paper first uses the English Chinese bilingual joint encoding input method based on special separators to achieve cross language semantic joint modeling. Then, this paper uses BERT to complete bilingual contextual semantic encoding, and then uses BiLSTM to enhance long-range time-dependent features and capture consistency constraints of the same term in different sentences. Finally, CRF ensures continuity and consistency across sentence boundaries through label transfer constraints, and combines transfer learning with domain fine-tuning to achieve adaptation in the cultural heritage field. The results showed that in the evaluation of error fragment level, the F1 score of the model was 92.8%; F1 achieved scores of 93.12%, 91.47%, and 90.86% in detecting mistranslations, omissions, and inconsistencies in terminology, respectively; while the illegal tag transfer rate had dropped to 0.03%. Research has shown that the proposed model has high structural stability and cross linguistic semantic discrimination ability in the task of detecting translation errors in cultural heritage terminology.

## 1 Introduction

Cultural heritage terminology is a core semantic unit in cross-cultural communication, and its translation quality directly affects the accuracy of cultural information transmission and the effectiveness of international communication. Because cultural heritage terminology carries deep cultural connotations such as history, aesthetics, and religion, there are common problems of conceptual mapping imbalance and cultural semantic deviation in English-Chinese translation (Jiang et al., 2022). With the continuous expansion of international cultural exchange, the number of bilingual texts in the field of cultural heritage has grown rapidly, and traditional manual proofreading methods are no longer sufficient to meet the quality assurance needs of high-frequency professional translations in terms of efficiency and consistency. The development of natural language processing and deep learning technologies has promoted research on automatic translation error detection, but there is still a lack of targeted structured detection methods for translation errors in cultural heritage terminology. Therefore, constructing an intelligent translation error detection model for cultural heritage terminology has significant research value and application significance (Zhang, 2023; Huang, 2024).

Current research on translation error detection mainly faces three difficulties. Cultural heritage terminology has a high degree of cultural load, and the semantics of the terminology often depend on specific historical and cultural contexts. Traditional lexical-level matching methods are difficult to identify deep cultural mistranslations that are “similar in form but different in meaning” (Chen and Zhou, 2024; Dalal-Clayton and Rutherford, 2024). There are obvious long-distance term dependencies within cultural heritage texts, and the translation of the same term in a text needs to be consistent. However, models based on local contexts have difficulty capturing cross-sentence term consistency constraints (Naveen and Trojovský, 2024; Song, 2023). The scale of high-quality bilingual error-annotated corpora in the field of cultural heritage is limited, and there are subjective differences in human annotation standards, which makes deep learning models prone to insufficient generalization ability and unstable error boundaries in domain scenarios (Klie et al., 2023; Saunders, 2022).

Existing translation error detection methods mainly fall into three categories: statistical machine learning models, neural network sequence models, and pre-trained language models. Traditional conditional random fields and support vector machines rely on manual feature construction, which has limited ability to express deep semantic relationships (Li et al., 2022). Recurrent neural networks such as BiLSTM improve the ability to extract sequence features through context modeling, but lack a direct modeling mechanism for cross-linguistic semantic correspondences (Gui and Xiao, 2024; Xu et al., 2024). Pre-trained language models such as BERT enhance the ability to represent contextual semantics through large-scale corpus pre-training, but the label-by-label independent classification method is prone to error boundary breaks and illegal label jumps (Ma et al., 2023). Existing research is mostly geared toward general translation quality assessment tasks, and lacks specific modeling for cultural semantic misalignment and terminology consistency issues in cultural heritage terminology.

To address the problems of difficulty in identifying deep semantic shifts, difficulty in modeling long-distance terminology dependencies, and unstable label structures in the translation of cultural heritage terminology, this paper constructs an automatic error detection model for the translation of cultural heritage terminology based on BERT-BiLSTM-CRF. This study defines translation error detection as a character-level BIO sequence labeling task and uses a bilingual concatenation structure of “[CLS] English original [SEP] Chinese translation [SEP]” to establish cross-lingual semantic associations. The model utilizes BERT for bilingual contextual semantic encoding, BiLSTM to enhance long-distance temporal dependency features, and CRF to achieve global structured decoding through label transfer constraints. The study combines transfer learning and domain fine-tuning to adapt the model to the characteristics of the cultural heritage domain and to enable it to discriminate between cross-sentence terminology consistency and cultural semantic shifts.

## 2 Construction of translation error detection model for cultural heritage terminology

### 2.1 Formalization of the translation error detection task

The core of error detection in the English-Chinese translation of cultural heritage terminology lies in identifying erroneous segments in the translation caused by cultural semantic shifts, imbalances in concept mapping, and inconsistencies in terminology systems, and jointly determining error boundaries and error types (Del Curto and Turrina, 2023; Lee et al., 2023). Traditional translation quality assessment tasks are usually completed using sentence-level score prediction or classification, lacking the ability to precisely locate error positions. However, errors in the translation of cultural heritage terminology are often concentrated in local terminological expressions, and their determination depends on the cultural semantic relationships of terms in the context (Jung et al., 2023; Guerreiro et al., 2024). Therefore, this paper defines this task as a character-level sequence labeling task for Chinese translations, achieving unified modeling of error positions, error boundaries, and error types by assigning corresponding labels to each character in the translation (Marier et al., 2025).

In the task formalization process, let the English original sequence be represented as $X^{en}={\text{x}}_{1}^{\text{en}},{\text{x}}_{2}^{\text{en}},\ldots ,{\text{x}}_{\text{m}}^{\text{en}}$, and the Chinese translation sequence be represented as $X^{zh}={\text{x}}_{1}^{\text{zh}},{\text{x}}_{2}^{\text{zh}},\ldots ,{\text{x}}_{\text{n}}^{\text{zh}}$. The goal of the model is to predict the corresponding label sequence *Y* = y_1_, *y*_2_, …, *y*_*n*_ for each character in the Chinese translation, given the bilingual input sequence, where *y*_*i*_ represents the label category corresponding to the *i*-th Chinese character, and *n* represents the length of the Chinese translation characters. The label set is constructed using the BIO annotation mechanism to simultaneously describe the boundary and error type information of error segments.

Errors in the translation of cultural heritage terms have boundary continuity and type consistency. The BIO structure can distinguish the beginning, interior, and non-error regions of error segments, thereby enhancing the model's ability to express complex error structures (Huang et al., 2023; Fan et al., 2023). To ensure the uniformity of structural expression for different error types, this paper constructs a multi-category BIO label system based on the main error patterns. In this system, MIS (Mistranslation) represents a terminology mistranslation error; Omission (OMI) represents a terminology omission error; Inconsistency (INC) represents a terminology inconsistency error. The label prefix B (Begin) indicates the starting position of the erroneous segment; I (Inside) indicates the internal position of the erroneous segment; and O (Outside) indicates a non-erroneous region. This labeling system simultaneously functions as error classification and boundary constraints, enabling subsequent sequence models to learn the structural transition relationships between different labels. Table 1 uniformly defines the structural meaning and task function of each label, providing standardized supervision objectives for BERT semantic encoding, BiLSTM temporal modeling, and CRF global decoding.

**Table 1:** Translation error BIO labeling system.

Label	Meaning of label	Error type	Sequence role
B-MIS	Start of mistranslation segment	Terminology mistranslation	Marks the beginning of an error boundary
I-MIS	Inside mistranslation segment	Terminology mistranslation	Maintains continuity of the error segment
B-OMI	Start of omission segment	Terminology omission	Marks the beginning of a missing segment
I-OMI	Inside omission segment	Terminology omission	Maintains continuity of the missing segment
B-INC	Start of inconsistency segment	Terminology inconsistency	Marks the beginning of a terminology conflict
I-INC	Inside inconsistency segment	Terminology inconsistency	Maintains continuity of the conflict segment
O	Non-error position	Correct translation	Marks normally translated characters

The BIO labeling system in Table 1 transforms the unstructured translation error problem into a standard sequence labeling problem, shifting the model training objective from traditional overall quality judgment to fine-grained character classification. B-class labels define the starting boundaries of error segments; I-class labels maintain the structural continuity within the same error segment; O-class labels distinguish normal translation regions. This structure not only improves the model's ability to locate complex error segments but also provides a clear state transition basis for global path constraints in subsequent CRF layers.

To further enable the computationalization of translation error detection, this paper models the character-level label prediction process as a conditional probability prediction problem. Given a bilingual input sequence, the model jointly predicts the label corresponding to the current character based on the contextual semantic information of the current position and the state of the preceding labels (Wang et al., 2023; Li et al., 2023). This modeling approach allows label prediction to move beyond single-character independent classification, learning the dependencies between labels across the entire label sequence, thus avoiding error boundary breaks and illegal label jumps, and providing a probabilistic basis for global structured decoding in subsequent CRF layers.

### 2.2 Design of bilingual semantic fusion model

Error detection in the translation of cultural heritage terms relies not only on local lexical information in the translation but also on the cross-linguistic semantic correspondence between source language terms and target language expressions (Sherborne et al., 2023; Giunchiglia et al., 2023). Cultural heritage terms carry a large amount of historical and cultural information, and the semantic meaning of the same term varies significantly in different contexts. Relying solely on the Chinese translation for error judgment can easily overlook the semantic constraints of the original text, making it difficult for the model to identify cultural concept misalignment and deep semantic shifts (Wang and Jiang, 2024; Deng et al., 2025). Therefore, this paper constructs a bilingual semantic fusion model. By uniformly encoding the English original and the Chinese translation, a term-level cross-linguistic association is established in a shared semantic space, and long-distance contextual dependency information is further strengthened, thereby improving the semantic discrimination ability of cultural heritage term translation error detection.

To establish direct semantic interaction between the source and target languages, this paper uses a bilingual sequence concatenation method to construct the model input structure (Dang et al., 2023). The model input can be represented as:


$$\begin{array}{l} X=\left[CLS\right]⊕X^{en}⊕\left[SEP\right]⊕X^{zh}⊕\left[SEP\right] \end{array}$$ (1)

In **Formula 1**, ⊕ represents the sequence concatenation operation; [*CLS*] represents the sequence start marker; [*SEP*] represents the sentence segment separation marker; *X* represents the final bilingual joint sequence input into the model. This input structure maps the original English text and the Chinese translation to a unified encoding space, enabling the model to directly establish cross-lingual semantic dependencies during the encoding process, thereby enhancing the perception ability of term translation correspondence.

After the bilingual input sequence enters BERT, it performs contextual semantic encoding through a multi-layer bidirectional Transformer structure. BERT uses a self-attention mechanism to dynamically aggregate global contextual information, enabling a fine-grained semantic alignment relationship between English terms and Chinese translations (de Varda and Marelli, 2023). Let the hidden state sequence output by BERT be:


$$\begin{array}{l} H=BERT\left(X\right) \end{array}$$ (2)

In **Formula 2**, *H* = h_1_, h_2_, …, h_n_ represents the context semantic representation sequence output by BERT, and *h*_*i*_ represents the dynamic semantic vector corresponding to the *i*-th Chinese character.

Since translation errors of cultural heritage terms often rely on long-distance context constraints and intra-textual terminology consistency, this paper introduces BiLSTM after BERT to further enhance temporal dependency information (Li et al., 2022). BiLSTM performs cyclic modeling of the semantic sequence from both forward and backward directions, and fuses the bidirectional hidden states to obtain the enhanced context representation:


$$\begin{array}{l} \tilde{h_{i}}=\left[\vec{h_{i}};\overset{⃖}{h_{i}}\right] \end{array}$$ (3)

In **Formula 3**, $\vec{h_{i}}$ represents the hidden state of the forward LSTM at position *i*; $\overset{⃖}{h_{i}}$ represents the hidden state of the backward LSTM at position *i*; $\tilde{h_{i}}$ represents the feature vector after fusing bidirectional temporal information; “;” represents the vector concatenation operation. The semantic representation enhanced by BiLSTM retains both cross-linguistic contextual information and long-distance dependency features, enabling the model to form a more stable discrimination ability against terminology semantic misalignment, contextual conflict, and translation inconsistency.

**Figure 1:**
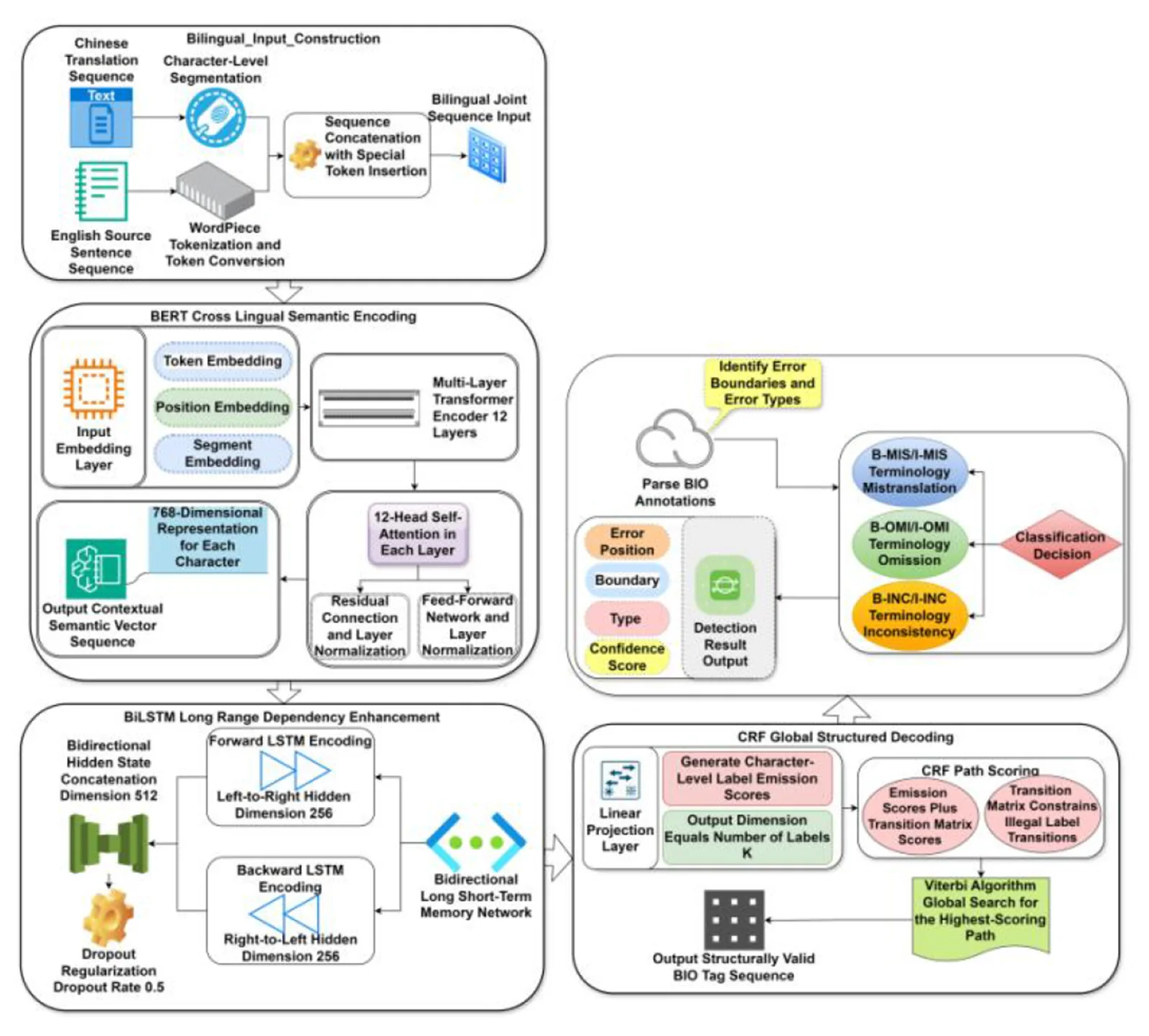
Framework for error detection in cultural heritage terminology translation.

The model in this paper uniformly describes the relationship between bilingual input, cross-language encoding, temporal reinforcement and structured output. Its structure reflects the hierarchical connection between BERT, BiLSTM, and CRF, and reflects the special requirements of cultural heritage terminology translation tasks for cross-language semantic fusion and long-range dependency modeling. The overall structure is shown in Figure 1.

Figure 1 illustrates the complete error detection process after bilingual input sequences undergo BERT cross-lingual semantic encoding, BiLSTM temporal dependency modeling, and CRF global structured decoding. The model constructs a unified cross-lingual representation space through joint modeling of the English source and Chinese translation, and retains semantic features at different granularities by combining WordPiece word segmentation and character-level segmentation. The Transformer multi-head self-attention mechanism is used to extract bilingual contextual association information, and BiLSTM further enhances the long-distance temporal dependency representation capability. Subsequently, CRF uses the label transition matrix to globally constrain and optimize the path of the BIO label sequence, reducing structural errors caused by illegal label transitions. Finally, it outputs the positional boundaries and confidence information corresponding to error types such as mistranslation, omission, and inconsistency of cultural heritage terminology, forming an end-to-end structured translation error detection model.

### 2.3 Global structured decoding based on CRF

Error detection in the translation of cultural heritage terms is a typical sequence labeling task. Error segments not only have clear boundary continuity, but also have strict label structure constraints. Mistranslation, omission, and inconsistency in cultural heritage terms usually appear in the form of continuous segments, and their label sequences must satisfy the BIO structure rules. If the traditional Softmax is used to classify each character independently, the model only completes the label prediction based on the current position features, and cannot establish global dependencies between labels. This can easily lead to problems such as illegal label jumps of type “O → I,” error boundary breaks, and inconsistent label types within the same error segment. Such structural errors directly affect the integrity and interpretability of the error localization results in the translation of cultural heritage terms. Therefore, this paper introduces Conditional Random Field (CRF) after the output layer of BiLSTM to perform global structured decoding of the label sequence (Rigouts Terryn et al., 2022).

After linear mapping, the context enhancement feature sequence output by BiLSTM is used to obtain the label emission score corresponding to each character. Let the size of the label set be *K*, then the emission vector corresponding to position *i* is expressed as:


$$\begin{array}{l} P_{i}=W\tilde{h_{i}}+b \end{array}$$ (4)

In **Formula 4**, $P_{i}\in R^{K}$ represents the label emission score vector corresponding to the *i*-th character; *W* represents the linear mapping weight matrix; *b* represents the bias vector. The emission score reflects the local probability tendency of the current position belonging to different label categories, but does not consider the structural dependencies between labels.

To jointly model the entire label sequence, CRF further introduces a label transition matrix *A* based on the emission score, used to describe the transition constraints between different labels. Let the predicted label sequence be *Y* = y_1_, y_2_, …, y_n_, and the context-enhanced feature sequence output by BiLSTM be *Z*. Then, the scoring function of CRF for the entire label path is defined as:


$$\begin{array}{l} \begin{array}{c} s\left(Z,Y\right)=\sum_{i=1}^{n}\left(A_{y_{i-1},y_{i}}+P_{i,y_{i}}\right) \end{array} \end{array}$$ (5)

In **Formula 5**, *s*(*Z, Y*) represents the global path score corresponding to the feature sequence *Z* and the label sequence *Y*; *A*_*y*_*i*−1_, *y*_*i*__ represents the transition score from label *y*_*i*−1_ to label *y*_*i*_; and *P*_*i*,_*y*__*i*__ represents the emission score when the *i*-th character is predicted as label *y*_*i*_.

Regarding translation errors in cultural heritage terminology, there are legitimate and illegitimate transfers among different BIO tags. Table 2 lists representative transfer rules directly related to the detection of mistranslations, omissions, and terminological inconsistencies, illustrating the constraint mechanism of CRF on the continuity of error boundaries and the consistency of tag structure. All other cross-type jumps not listed are considered illegitimate transfers according to BIO structural constraints, thus providing an explicit structural basis for the model to learn the boundaries of error segments and the continuity of tags.

**Table 2:** BIO tag transfer constraints.

Current label	Next label	Transition type	Structural meaning
B-MIS	I-MIS	Valid transition	Maintains continuity of mistranslation segment
I-MIS	I-MIS	Valid transition	Continues the mistranslation region
B-OMI	I-OMI	Valid transition	Maintains continuity of omission segment
B-INC	I-INC	Valid transition	Maintains continuity of terminology inconsistency
O	B-MIS	Valid transition	Start of a new error segment
O	I-MIS	Invalid transition	Missing start of error boundary
O	I-OMI	Invalid transition	Break in omission boundary

Table 2 shows the label transition relationships, reflecting the BIO structure's constraint requirements on the integrity of error boundaries. Legitimate transitions ensure the continuity and consistency of labels within the same error segment, while illegal transitions correspond to structural anomalies such as missing starting boundaries, error type breaks, and cross-type abnormal jumps. CRF learns the label transition matrix and assigns lower transition scores to illegal paths, thereby suppressing the generation of structurally invalid label sequences.

After obtaining the scores of all candidate paths, CRF calculates the conditional probability of the label sequence using global normalization:


$$\begin{array}{l} P\left(Y|Z\right)=\frac{\exp\left(s\left(Z,Y\right)\right)}{\sum_{Y^{\prime }\in Y_{Z}}\exp\left(s\left(Z,Y^{\prime }\right)\right)} \end{array}$$ (6)

In **Formula 6**, *P*(*Y*|*Z*) represents the conditional probability of the label sequence *Y* corresponding to the feature sequence *Z*; *Y*_*Z*_ represents the set of all possible label paths corresponding to the input sequence; *Y*′ represents any possible label sequence in the candidate label path set. CRF, by jointly modeling the probabilities of the entire path, transforms the model output from being limited to local optimal prediction of a single character into a global optimal search problem for the entire label path.

In the model training phase, the negative log-likelihood function is used as the optimization objective, and parameter learning is achieved by maximizing the probability of the true label path. In the prediction phase, the Viterbi algorithm is used to search for the label path with the highest score:


$$\begin{array}{l} Y^{*}=\arg s(Z,Y) \end{array}$$ (7)

In **Formula 7**, Y^*^ represents the optimal label sequence obtained by the final prediction. Viterbi decoding performs a global search on all possible paths through dynamic programming, and obtains the label sequence with the highest score while ensuring computational efficiency. After global constraints by CRF, the error detection results of cultural heritage terminology translation are further enhanced in terms of error boundary integrity, label type consistency and structural logical legality, providing a stable foundation for error fragment-level evaluation in subsequent experiments.

### 2.4 Model training and domain fine-tuning

The task of detecting translation errors in cultural heritage terminology is characterized by low resource availability. The scale of high-quality bilingual error-annotated corpora in the domain is limited, while deep neural network models have large parameter scales. If training is performed directly using random initialization, the model is prone to slow convergence, unstable semantic representation, and insufficient learning of domain features (Tonja et al., 2023; Liu et al., 2023). Cultural heritage terminology itself has strong professionalism and cultural dependence. Error judgment depends not only on the surface structure of language but also on the deep semantic relationships of terms in cross-linguistic contexts. Therefore, this paper adopts a training method combining transfer learning and domain fine-tuning. BERT parameters pre-trained on general corpora are used as the initialization basis, and end-to-end joint optimization is performed on cultural heritage terminology translation error-annotated data to gradually adapt the model to the characteristics and error patterns of cultural heritage terminology (Chen et al., 2024; Tran et al., 2024).

During the model training phase, the bilingual input sequence is processed by BERT, BiLSTM, and CRF to output the conditional probability of the label sequence. To increase the probability ratio of the true label path among all candidate paths, this paper uses the negative log-likelihood function as the overall optimization objective:


$$\begin{array}{l} L=-\log P\left(Y|Z\right) \end{array}$$ (8)

In **Formula 8**, *L* represents the model training loss function. Minimizing the loss function is essentially maximizing the true label path score, enabling the model to gradually learn the semantic features and label structure patterns in cultural heritage terminology translation errors.

Since the BERT parameters have already been pre-trained on a large-scale general corpus, their internal parameters contain rich linguistic knowledge and contextual semantic representation capabilities. Therefore, this paper adopts a holistic joint fine-tuning strategy to synchronously update the BERT, BiLSTM, and CRF parameters. Let the model parameter set be represented as:


$$\begin{array}{l} \Theta =\left\{{\text{Θ}}_{BERT},\Theta_{BiLSTM},\Theta_{CRF}\right\} \end{array}$$ (9)

In **Formula 9**, Θ_*BERT*_ represents the BERT encoding layer parameter set; Θ_*BiLSTM*_ represents the BiLSTM temporal modeling layer parameter set; Θ_*CRF*_ represents the CRF structured decoding layer parameter set. During training, all parameters are jointly optimized through the backpropagation algorithm, allowing general linguistic knowledge to gradually migrate to the semantic space of cultural heritage terminology.

To avoid overfitting under limited training samples, this paper introduces a Dropout mechanism after the BiLSTM output layer to randomly deactivate the output of some neurons (Lu et al., 2023). Let the Dropout output be represented as:


$$\begin{array}{l} \tilde{h}=m⊙h \end{array}$$ (10)

In **Formula 10**, *h* represents the BiLSTM output feature vector; *m* represents the random deactivation mask vector; ⊙ represents the element-wise multiplication operation; $\tilde{h}$ represents the feature representation after Dropout. This mechanism reduces the model's over-reliance on local features, enhancing its generalization ability in the cultural heritage terminology translation error detection task.

Model parameter updates are performed using the Adam optimization algorithm. Adam dynamically estimates the first and second moments of the gradient, adaptively adjusting the learning rate of different parameters to improve model training stability. The parameter update process is expressed as:


$$\begin{array}{l} \Theta_{t+1}=\Theta_{t}-\eta \cdot \frac{{\hat{m}}_{t}}{\sqrt{{\hat{v}}_{t}}+\epsilon } \end{array}$$ (11)

In **Formula 11**, Θ_*t*_ represents the model parameters at iteration *t*; η represents the learning rate; ${\hat{m}}_{t}$ represents the estimated first moment of the gradient; ${\hat{v}}_{t}$ represents the estimated second moment of the gradient; ϵ represents the smoothing term to prevent the denominator from being zero. The Adam optimization mechanism weakens the impact of gradient oscillations on model convergence stability during training, enabling the model to maintain a stable training state under low-resource domain data conditions.

**Figure 2:**
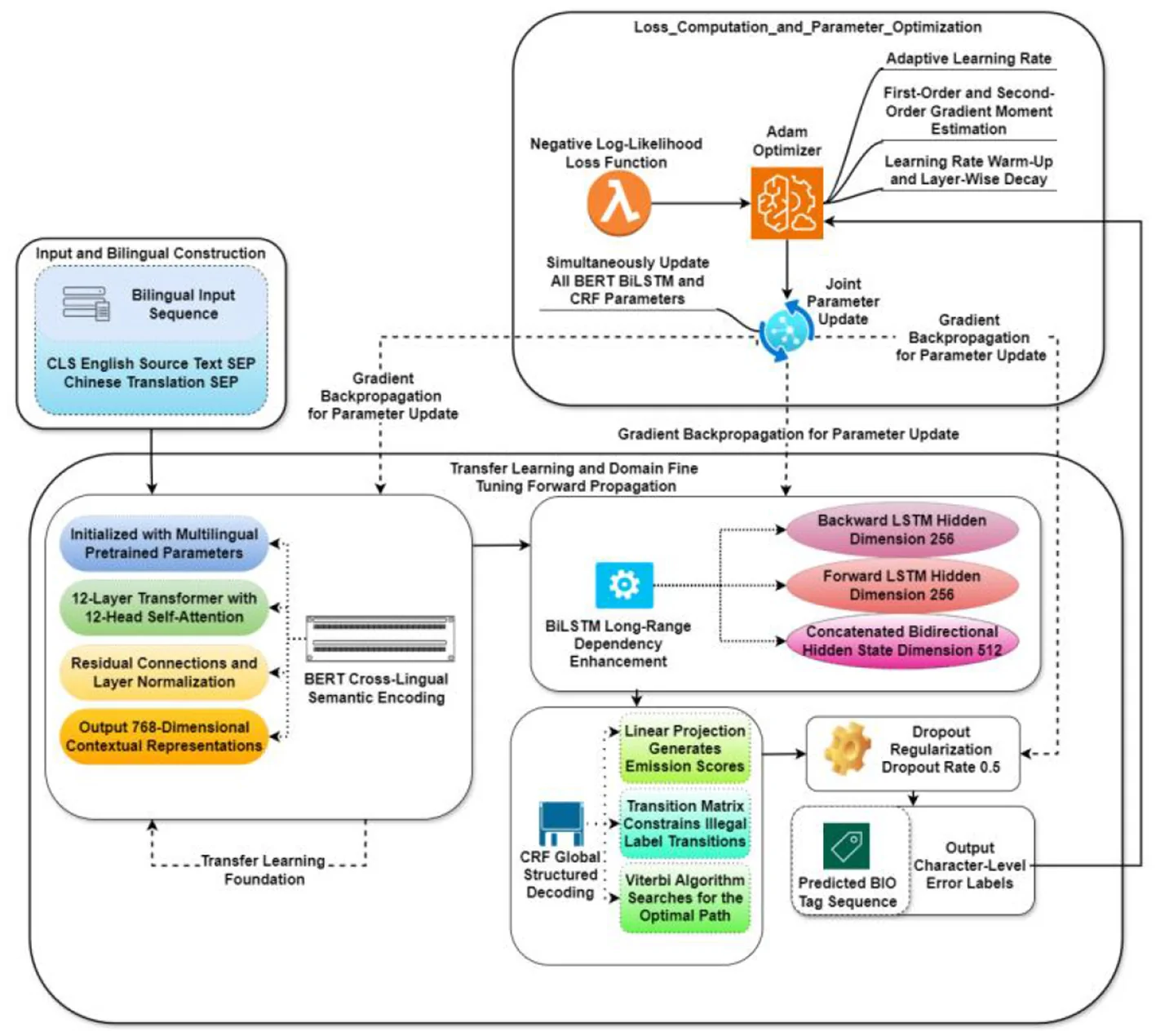
Schematic diagram of cross-lingual attention interaction mechanism in bilingual sequences.

To illustrate how bilingual concatenation input drives BERT to establish cross-linguistic term associations, Figure 2 illustrates the term-level interaction of self-attention heads within the encoding space “[CLS] English original text [SEP] Chinese translation [SEP]”: an attention interaction path is formed between English term lexical units and their corresponding Chinese translation characters, which enables the model to directly perceive the constraint relationship between the semantics of the source language terms and the expression of the target language during the encoding process.

Figure 2 illustrates the structural process of cross-lingual interaction paths formed by English terminology and Chinese translation characters under the BERT self-attention mechanism: the arrows indicate the direction of attention flow, and the [SEP] delimiter divides the bilingual sequence into two encoding segments: the source language and the target language. Figure 2 illustrates from a mechanistic perspective how the bilingual concatenation encoding structure provides a cross-lingual semantic foundation for subsequent BiLSTM temporal modeling and CRF structured decoding.

## 3 Experiment on error detection in translation of cultural heritage terminology

### 3.1 Dataset source and data preprocessing

The experimental data mainly comes from the publicly available bilingual texts in the UNESCO World Heritage Database and the WMT publicly available translation evaluation dataset. The UNESCO corpus and the WMT quality estimation corpus serve different purposes during data construction. UNESCO bilingual texts provide domain specific terminology and authentic translation contexts, while WMT annotations provide supervision for translation error identification. Domain terminology filtering is performed before corpus integration, and only bilingual sentence pairs containing standardized cultural heritage terminology are retained. All retained samples are converted into the same character level BIO annotation format to maintain annotation consistency throughout the merged corpus. The UNESCO World Heritage Database has long published World Heritage application texts, heritage introductions, protection descriptions, and historical backgrounds, etc., and the texts have a high density of cultural terms and cross-lingual semantic features. The experiment extracts sentences and aligns the English texts and corresponding Chinese translations in the database at the sentence level, and then converts them into UTF-8 encoding format. The preprocessing stage cleans HyperText Markup Language (HTML) tags, special characters, repeated spaces, and invalid segments to ensure structural consistency and semantic integrity of the two statements.

The translation error supervision data construction utilizes publicly released Automatic Post-Editing (APE) and Quality Estimation (QE) task data from WMT. Since the original WMT quality estimation annotations are provided at the word level, each annotated error span is first aligned with the corresponding bilingual sentence pair through word position matching. The aligned word boundaries are then projected onto the Chinese character sequence according to the bilingual alignment relationship. The first character within each error span is assigned the corresponding B label, the remaining characters are assigned the corresponding I label, and all other characters are assigned the O label. Continuous character spans are retained throughout the conversion process to preserve complete BIO boundary consistency. The dataset consists of machine translation results, manually edited text, and quality annotation information. The experiment uses the differences between the manually edited results and the original translations to generate error alignment information, and combines this with a cultural heritage terminology glossary to filter sentence pairs containing domain terms. After BIO conversion, all generated label sequences are automatically verified to detect missing beginning labels, illegal label transitions, inconsistent error categories within one segment, and duplicated boundaries. Samples failing the verification process are corrected before entering model training, ensuring complete structural consistency of all BIO annotations.

The cultural heritage terminology glossary is derived from publicly available terms in the World Heritage List, UNESCO cultural heritage subject classification vocabularies, and professional terminology entries in the Encyclopedia of China: Cultural Relics and Museums Volume. In the terminology standardization stage, English terms and Chinese translations are uniformly mapped, and synonymous and variant expressions are merged to reduce terminology noise interference. After filtering, only valid bilingual samples containing cultural heritage terms are retained. After terminology normalization, sentence pairs originating from different data sources follow the same terminology mapping rules and annotation criteria. This preprocessing procedure reduces differences caused by heterogeneous corpus sources and maintains consistent semantic representation across the final dataset. The final corpus is constructed after source-specific preprocessing rather than directly combining heterogeneous annotations, ensuring that different data sources follow the same annotation criteria before participating in model training.

In the text encoding stage, English text is segmented using WordPiece, and Chinese translations are segmented at the character level. Bilingual sequences are concatenated according to the format “[CLS] Original English [SEP] Chinese Translation [SEP],” and truncation and padding are performed based on the maximum input length. For sequences shorter than the maximum length, padding is added to the end, and an Attention Mask sequence of the same length as the input sequence is generated. In the Attention Mask, the positions corresponding to real words are assigned a value of 1, and the padding positions are assigned a value of 0, which is used to shield invalid padding information from interfering with the semantic modeling of the context during BERT self-attention calculation.

Due to the limited scale of mislabeled data in the cultural heritage field, the experiment performs data augmentation on the training set. Data augmentation is applied only after the original annotation process has been completed. Every augmented sample inherits the original error category and undergoes a second verification to ensure that terminology correspondence, error boundaries, and BIO labels remain unchanged. Samples failing semantic consistency verification are discarded before model training. The semantic consistency verification evaluates whether augmented sentences preserve the original meaning representation and terminology relations before entering the training process. The generated samples are compared with their corresponding original sentences in terms of cultural heritage term alignment, translation error category consistency, and contextual semantic coherence. Augmented sentences introducing semantic deviation or changing the original error characteristics are removed to prevent misleading supervision signals during model optimization. The augmentation strategy combines back-translation perturbation and terminology replacement. Back-translation perturbation uses a publicly available neural machine translation model to generate new samples that preserve semantics but have different expressions; terminology replacement is performed based on the cultural heritage terminology lexicon to replace similar terms, and the BIO label boundaries are adjusted simultaneously. The complete corpus is divided into training, validation, and test subsets according to a ratio of approximately seventy percent, fifteen percent, and fifteen percent. Data augmentation is applied only to the training subset, while the validation subset and test subset retain the original annotations to preserve an unbiased evaluation process.

### 3.2 Experimental environment and parameter settings

The experiment is conducted using the PyTorch framework for model construction and training. The BERT encoding layer is implemented using the open-source Hugging Face Transformers. The experimental environment is deployed on a Linux server platform, using an NVIDIA Tesla V100 GPU for training and inference computation. The Compute Unified Device Architecture (CUDA) and CUDA Deep Neural Network library (cuDNN) versions are consistent with PyTorch to ensure stable operation of the Transformer and BiLSTM computation processes.

The model encoding layer uses the BERT-Based Multilingual Cased pre-trained model as initial parameters. This model is pre-trained based on a multilingual wiki corpus and possesses unified cross-lingual semantic representation capabilities. The BERT hidden layer dimension is set to 768; the number of self-attention heads is set to 12; the number of Transformer encoding layers is set to 12. The BiLSTM adopts a bidirectional single-layer structure, with the hidden state dimension set to 256. The output features are dropped and then input into the CRF layer for structured decoding, with a Dropout ratio set to 0.5.

The AdamW optimization algorithm is used during model training, and a linear learning rate warm-up mechanism is introduced. The BERT encoding layer, BiLSTM, and CRF layers employ a hierarchical learning rate strategy. The BERT part uses a lower learning rate to maintain pre-trained semantic stability, while the BiLSTM and CRF layers share a higher learning rate to accelerate domain feature adaptation and label transfer parameter convergence. The training batch size is set based on GPU memory capacity, and the maximum length of the input sequence is uniformly limited to 256 characters. Sequences exceeding this length are truncated, and sequences below the required length are padded.

To ensure experimental reproducibility, the core parameters during model training are uniformly controlled, as shown in Table 3.

**Table 3:** Model training parameter configuration.

Parameter name	Parameter setting	Parameter type	Parameter function
BERT hidden size	768	Model structure	Bilingual semantic representation
Number of transformer layers	12	Model structure	Deep contextual encoding
Number of attention heads	12	Model structure	Multi-head semantic interaction
BiLSTM hidden size	256	Sequential modeling	Long-range dependency modeling
Dropout rate	0.5	Regularization	Prevents overfitting
Batch size	16	Training control	Mini-batch gradient update
Max sequence length	256	Input control	Standardizes bilingual sequence length
BERT learning rate	2 × 10^−5^	Optimization	Fine-tuning pretrained parameters
BiLSTM learning rate	1 × 10^−3^	Optimization	Sequential layer parameter update
Number of epochs	20	Training control	Iterative model optimization

Table 3 shows a unified parameter setting configured to meet the bilingual semantic modeling requirements of the cultural heritage terminology translation error detection task. BERT parameters control cross-linguistic contextual semantic representation capabilities; BiLSTM parameters enhance long-range temporal dependency information; and the CRF structure fulfills global label constraints. The hierarchical learning rate balances the update intensity between pre-training knowledge retention and domain feature learning, while training control parameters ensure consistency in the model training process under different experimental conditions.

During the experimental evaluation phase, two evaluation granularities are used: character-level and error fragment-level. Character-level evaluation is based on the statistical classification performance of single-character label prediction results; error fragment-level evaluation requires that predicted fragments and true error fragments be completely consistent in terms of boundaries and error types. Evaluation metrics include precision, recall, and F1 score. Precision measures the proportion of true errors among predicted errors; recall measures the proportion of true errors detected; and the F1 score is the harmonic mean of precision and recall, used to comprehensively reflect the overall detection performance of the model. All models are trained and tested under the same data partitioning and parameter conditions to ensure consistency of experimental results.

Besides detection accuracy, practical deployment efficiency is also an important consideration for translation quality assurance systems. Since the proposed model is intended for automatic inspection of bilingual translation results, inference efficiency, computational resource consumption, and model complexity are evaluated under the same hardware environment described above. The deployment evaluation is performed using a batch size of one to simulate online translation quality inspection, thereby reflecting the computational characteristics of the model during practical inference. The corresponding deployment performance statistics are summarized in Table 4.

**Table 4:** Model deployment performance statistics.

Metric	Measured value	Test condition	Description
Number of trainable parameters	178.6 M	BERT-BiLSTM-CRF	Total model size
GPU memory usage	2.84 GB	NVIDIA Tesla V100	Peak GPU memory consumption during inference
Average inference latency	18.6 ms/sentence	Batch Size = 1	Average inference time per sentence
Inference throughput	53.8 sentences/s	Batch Size = 1	Number of sentences processed per second
Model initialization time	2.31 s	Linux server	Time required to load the model before inference

Table 4 shows that the proposed model maintains moderate computational resource consumption while preserving high detection accuracy. GPU memory usage remains within the capacity of commonly used deep learning computing platforms, and the average inference latency is maintained at the millisecond level for individual bilingual sentence pairs. The measured throughput further indicates that the proposed model satisfies the efficiency requirements of continuous translation quality inspection and provides practical support for deployment in automatic translation quality assurance scenarios.

### 3.3 Comparison models and evaluation metrics

To evaluate the performance of the proposed model in the task of detecting errors in the English-Chinese translation of cultural heritage terminology, a baseline model comparison system and a structure ablation experiment system are constructed. All models are trained and tested under the same dataset partitioning, uniform training epochs, and consistent evaluation metrics. A character-level BIO tagging system and bilingual concatenation input format are uniformly adopted to ensure the comparability of experimental results.

The baseline models are set as three classes: BiLSTM-CRF, BERT-Softmax, and BERT-CRF. The BiLSTM-CRF model uses static word vector input, extracts contextual temporal features through BiLSTM, and performs label decoding using CRF, to observe the performance of traditional sequence labeling structures in detecting errors in the translation of cultural heritage terminology. The BERT-Softmax model retains the cross-lingual semantic encoding structure of BERT and uses Softmax to independently predict character labels, to analyze prediction behavior when global label constraints are lacking. The BERT-CRF model directly connects the CRF structure after the BERT output layer, and the impact of long-distance temporal dependency reinforcement mechanisms on the error detection task by removing BiLSTM is observed.

Considering that error detection in cultural heritage terminology translation involves factors such as cross-linguistic semantic alignment, long-range contextual dependencies, and label structure constraints, the experiment further designs ablation experiments to independently remove core components of the model. After removing BiLSTM, the model directly uses BERT output for CRF decoding to analyze the effect of temporal dependency reinforcement mechanisms on the semantic continuity of terms. After removing CRF, the model uses Softmax instead of structured decoding to analyze the impact of global label constraints on the integrity of error boundaries. After removing BERT, the model no longer uses pre-trained cross-linguistic semantic encoding structures, retaining only BiLSTM and CRF to complete Chinese translation sequence modeling, to analyze the role of pre-trained cross-linguistic semantic representations in error detection in cultural heritage terminology translation.

During the experiment, all models use a fixed random seed for parameter initialization and training control, and employ a uniform batch size and optimizer parameter settings, retaining only structural differences between models. During model evaluation, both character-level and error fragment-level prediction results are recorded, and statistical analyses are performed on three types of errors: mistranslation, omission, and inconsistency in terminology. Predicted outputs are uniformly converted into BIO label sequences, and error fragment matching is performed based on label boundaries and label types.

To analyze the impact of model structure on label validity, the experiment uniformly statistically analyzes illegal “O → I” jumps, error type breaks, and missing boundaries in the predicted sequences, and calculates the proportion of illegal label transfers. In the cross-lingual semantic modeling capability analysis, the attention matrix output from the last Transformer layer of BERT is extracted, and the attention weight distribution between English term positions and Chinese translation positions is statistically analyzed to construct a cross-lingual semantic heatmap. All attention results are normalized and mapped to a uniform color space to ensure consistency in attention distribution results across different models.

## 4 Results

### 4.1 Overall performance comparison of different models

**Figure 3:**
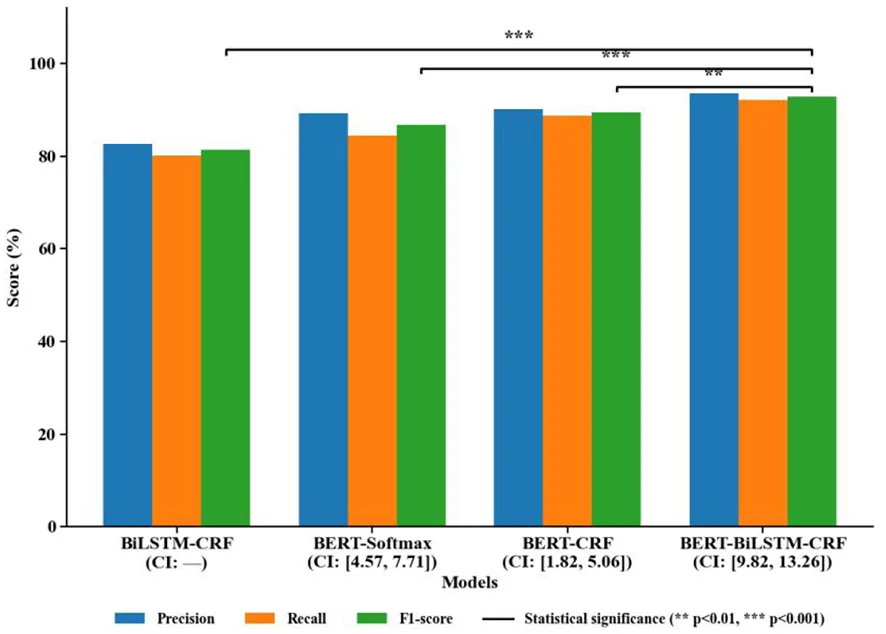
Comparison of error fragment detection performance of various models. **represents *p* < 0.01, ***represents *p* < 0.001.

To verify the overall effectiveness of the constructed BERT-BiLSTM-CRF model in the task of detecting translation errors in cultural heritage terminology, the experiment compares the proposed model with three baseline models—BiLSTM-CRF, BERT-Softmax, and BERT-CRF—under the same dataset partitioning, unified training epochs, and consistent evaluation metrics. The contribution of each component to the overall detection performance is analyzed from three dimensions: BERT cross-linguistic semantic modeling, BiLSTM long-range dependency enhancement, and CRF global structural constraints. Error fragment-level precision, recall, and F1 score are used as the core evaluation metrics. The overall performance comparison results of each model are shown in Figure 3. To further evaluate the statistical reliability of the performance differences, Bootstrap resampling was performed on the error fragment level. The confidence intervals and significance labels presented in Figure 3 provide statistical support for the comparison results between the proposed model and the baseline models.

In Figure 3, the model presented in this paper achieves an F1 score of 92.8%, the best performance among all models compared. BiLSTM-CRF's F1 score is approximately 81.3%, a difference of 11.5 percentage points compared to the model presented in this paper. This gap mainly stems from the insufficient expressive power of static word vectors for cross-linguistic semantics of cultural heritage terms. It lacks direct modeling of the semantic correspondence between English terms and their Chinese translations, thus limiting its ability to identify deep cultural semantic shifts. BERT-Softmax's F1 score improves to 86.7% after introducing pre-trained semantic representations, but due to the lack of label structure constraints in its character-by-character independent classification mechanism, illegal label jumps are not effectively suppressed, resulting in frequent breakages in error boundaries, and a significant difference between its recall and precision. The BERT-CRF model without BiLSTM achieves an F1 score of 89.4%, 3.4 percentage points lower than the model presented in this paper. This indicates that relying solely on BERT contextual semantic encoding without explicit reinforcement of long-distance temporal dependencies significantly weakens the model's ability to capture cross-sentence terminology consistency constraints, and it remains insufficient in judging the coherence of discourse-level terminology in cultural heritage texts. The tiered performance differences among the three baseline models underscore the necessity of the synergistic combination of BERT cross-linguistic encoding, BiLSTM temporal modeling, and CRF global decoding for detecting translation errors in cultural heritage terminology. The Bootstrap analysis further supports the reliability of the observed performance differences. The confidence intervals of all pairwise comparisons remain above zero, and the significance labels shown in Figure 3 indicate that the performance improvements of the proposed model over all baseline models remain stable across repeated resampling. The end-to-end joint model constructed in this paper achieves optimal detection performance under the condition of effective integration of all components.

### 4.2 Detection capability for different error types

**Figure 4:**
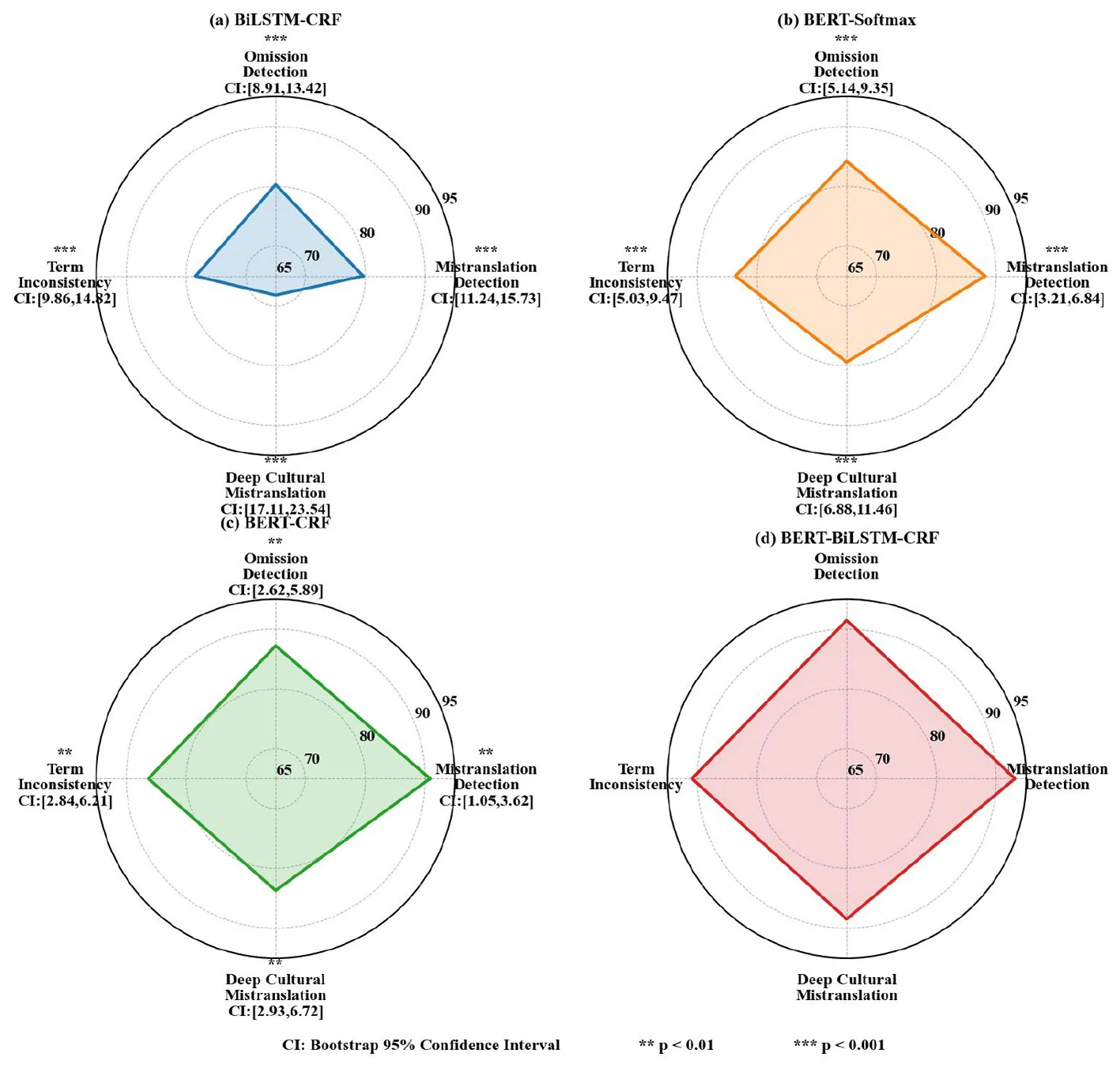
Comparison of error detection capabilities of different models. **(a)** Distribution of error detection capabilities of BiLSTM-CRF for different error types. **(b)** Distribution of error detection capabilities of BERT-Softmax for different error types. **(c)** Distribution of error detection capabilities of BERT-CRF for different error types. **(d)** Distribution of error detection capabilities of BERT-BiLSTM-CRF for different error types. **represents *p* < 0.01, ***represents *p* < 0.001.

Errors in the translation of cultural heritage terminology exhibit significant differences in type distribution and semantic causes. Mistranslation, omission, and inconsistency in terminology vary in their dependence on cross-linguistic semantic modeling, long-range contextual dependencies, and label structure constraints. A single overall performance metric cannot fully reflect the model's detection capability under different error patterns. Therefore, experiments are conducted between the proposed model and three baseline models (BiLSTM-CRF, BERT-Softmax, and BERT-CRF) to statistically analyze the F1 score of each error type at the error fragment level. Furthermore, targeted analysis is performed on the detection performance in deep cultural mistranslation scenarios. The distribution of detection capabilities for each model across different error types is shown in Figure 4, which also presents the confidence intervals and significance labels from Bootstrap resampling on the error fragment level for all pairwise comparisons to further examine the reliability of the performance differences.

In Figure 4a, BiLSTM-CRF has the lowest scores in all four metrics, with a deep cultural mistranslation detection F1 score of only 68.2%. The fundamental reason is that static word vectors cannot capture the dynamic semantic shift of culturally loaded words in cross-linguistic contexts, resulting in a large number of missed detections of deep cultural mistranslations of the “similar in form but different in meaning” type. This shortcoming also restricts the overall performance of mistranslation detection. In Figure 4b, BERT-Softmax improves in all four metrics after introducing pre-trained semantic representations, with the deep cultural mistranslation detection F1 score rising to 79.4%. However, this model shows a significant gap between precision and recall in all four types of errors. This is because the character-by-character independent classification mechanism cannot form a coherent constraint on the boundaries of continuous error segments, and the fragmentation of labels within segments directly lowers the recall statistics. In Figure 4c, the F1 score for deep cultural mistranslation detection by BERT-CRF is 83.7%, while the F1 score for omission detection is 4.27 percentage points lower than that of the model in this paper. This is because the model fails to capture the temporal dependencies of missing components across sentences after removing BiLSTM. When omissions are distributed at distant locations within a text, BERT's Transformer attention mechanism alone is insufficient to stably locate the missing boundaries. In Figure 4d, the F1 scores of the model in this paper are improved by 2.32, 4.27, 4.56, and 4.8 percentage points compared to BERT-CRF, respectively. The Bootstrap analysis further confirms the stability of these improvements across all error categories. The confidence intervals of all pairwise comparisons remain above zero, and the significance labels presented in Figure 4 indicate consistent performance differences between the proposed model and the three baseline models under repeated resampling. The largest improvement is observed in deep cultural mistranslation detection, followed by mistranslation detection, terminology inconsistency detection, and omission detection, indicating that the joint modeling strategy maintains stable advantages across different translation error patterns. Terminology inconsistency detection performs worse than mistranslation and omission detection in all four models. This is directly related to the fact that the identification of this error type highly depends on the text-level translation consistency constraints, while the local modeling window of the sequence labeling framework has inherent limitations. The model in this paper achieves optimal coverage in all four error detection types through the synergistic effect of BERT cross-linguistic semantic encoding, BiLSTM temporal dependency reinforcement, and CRF global label constraints. The Bootstrap verification further shows that the performance advantages remain consistent across repeated resampling, supporting the robustness of the joint modeling structure for detecting multiple types of translation errors in cultural heritage terminology.

### 4.3 Ablation experiments

**Figure 5:**
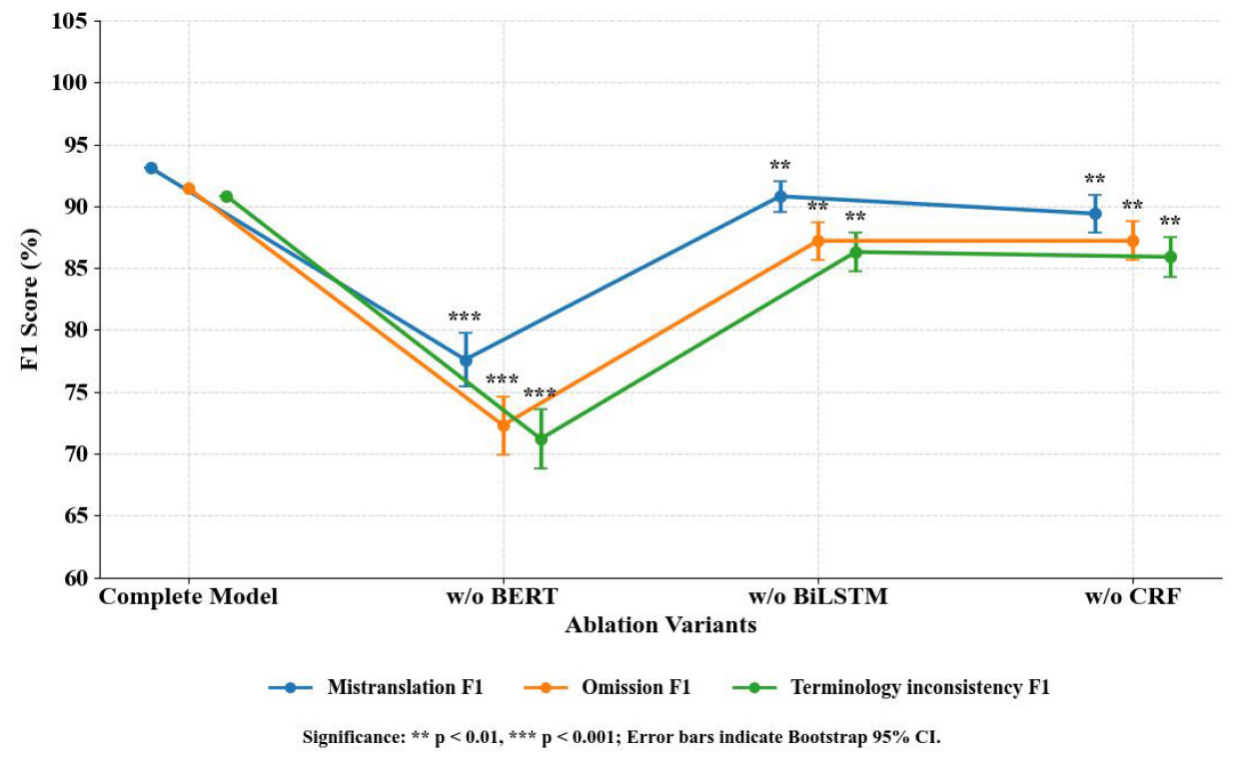
Comparison of F1 scores after removing each component in the ablation experiment. **represents *p* < 0.01, ***represents *p* < 0.001.

The overall performance comparison has verified the comprehensive advantages of the proposed model. However, the independent contribution of each component to the final detection performance and its role mechanism in the task of detecting translation errors in cultural heritage terminology still need further clarification. Therefore, under the condition of unified dataset partitioning and training, the three core components—BERT, BiLSTM, and CRF—are removed from the complete model in turn to construct three ablation variants. The F1 scores at the error fragment level are compared with the complete model. Figure 5 presents both the performance degradation of each ablation variant relative to the complete model and the corresponding confidence intervals and significance labels from Bootstrap resampling on the error fragment level, used to assess the reliability of the degradation after removing different components.

As shown in Figure 5, removing BERT results in the largest decrease in the model's F1 score, averaging approximately 18.1 percentage points lower. This directly reflects the core supporting role of pre-trained cross-lingual semantic encoding in detecting translation errors of cultural heritage terms. Without the contextual semantic representation accumulated through large-scale multilingual corpus pre-training, the model's discrimination of English-Chinese terminological semantic correspondences degenerates into reliance on shallow lexical features, failing to identify deep shifts in culturally loaded words in cross-lingual mappings. Removing BiLSTM results in an average decrease of approximately 3.7 percentage points in the F1 score, a relatively mild decrease. However, the performance degradation is significantly concentrated in the two types of errors: omissions and terminological inconsistencies. This is because while BERT's self-attention mechanism covers the global context, its explicit modeling ability for sequential temporal direction is inferior to that of bidirectional recurrent structures. In scenarios involving long-distance textual dependence in cultural heritage texts, the fusion and enhancement of forward and backward temporal features by BiLSTM cannot be completely replaced by Transformer attention. After removing CRF, the F1 score decreases by an average of about 4.3 percentage points. The Bootstrap verification shows that the confidence intervals of all comparisons remain above zero, and the significance labels presented in Figure 5 indicate stable performance differences between the complete model and each ablation variant under repeated resampling. The largest performance degradation is observed after removing BERT, while removing BiLSTM and CRF also leads to consistent reductions across different error categories. The root cause is that the lack of label transition constraints in the Softmax independent classification method directly leads to frequent illegal jumps in the “O → I” class, and the broken boundaries of erroneous segments significantly reduce the success rate of segment-level matching. The ablation results together with the Bootstrap verification show that BERT provides the primary contribution to the overall detection performance, while the observed performance degradation remains consistent across repeated resampling. BiLSTM and CRF jointly improve temporal dependency modeling and structured sequence decoding, and the Bootstrap verification indicates that the performance differences produced by removing either component remain consistent under repeated resampling. The removal of any individual component leads to sustained reductions in translation error detection performance, supporting the coordinated contribution of semantic representation learning, temporal dependency modeling, and structured sequence decoding within the proposed framework.

### 4.4 Effectiveness of CRF global constraints

**Figure 6:**
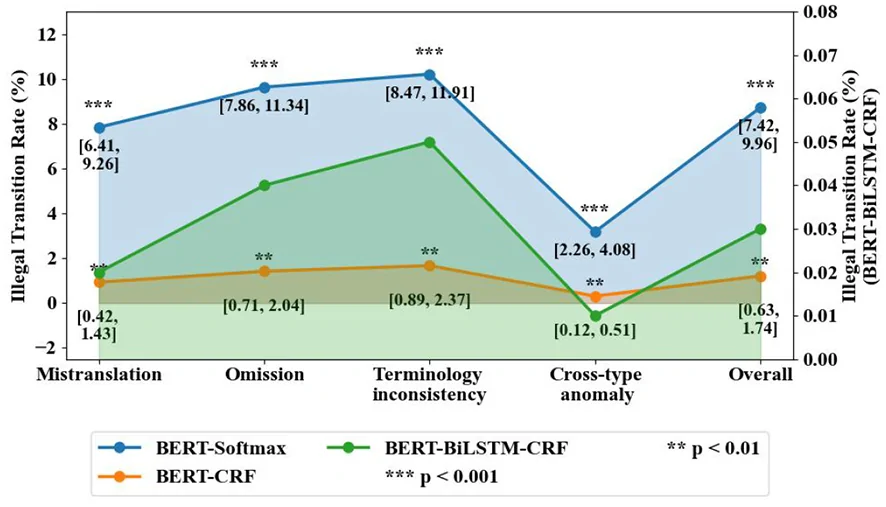
Comparison of illegal transfer distribution of BIO labels in different models. **represents *p* < 0.01, ***represents *p* < 0.001.

Fragment-level performance metrics such as the F1 score reflect the overall detection performance of the model, while the structural legality of the label sequence directly determines the reliability of error boundary localization; the two are not equivalent. To quantify the actual contribution of the CRF global constraint mechanism to the structural legality of BIO labels, experiments are conducted to statistically analyze the illegal BIO label transitions in the predicted sequences of the proposed model, BERT-Softmax, and BERT-CRF on the test set. The proportions of illegal transitions in mistranslated segments, omitted segments, inconsistent terminology segments, and cross-type abnormal jumps are calculated, respectively. The overall illegal label transition rate is used as a comprehensive evaluation index. Figure 6 shows the distribution of illegal BIO label transitions for each model, along with the confidence intervals and significance labels from Bootstrap resampling on the predicted label sequences for all pairwise comparisons to evaluate the reliability of the differences among models.

Figure 6 shows that the overall illegal transfer rate of the model presented in this paper is reduced to 0.03%, while the illegal transfer rate of BERT-Softmax is approximately 8.72%, and that of BERT-CRF is approximately 1.21%. The Bootstrap verification further indicates that the confidence intervals of all pairwise comparisons remain above zero, and the significance labels presented in Figure 6 show consistent reductions in illegal transition rates under repeated resampling. The significantly higher illegal transfer rate of BERT-Softmax is due to the fact that the character-by-character independent classification mechanism of Softmax completely severs the structural dependency between adjacent labels. The model cannot perceive the state of the preceding label during the prediction process, resulting in the frequent occurrence of typical illegal paths such as “O → I-MIS” and “O → I-OMI” in continuous term segments. The phenomenon of broken error boundaries is concentrated in omission and term inconsistency errors. Although BERT-CRF compresses the overall illegal transfer rate to 1.21% by introducing a label transfer matrix, due to the lack of explicit modeling of long-distance temporal dependencies by BiLSTM, the model's learning of label transfer constraints at cross-sentence term segments is insufficient, and some cross-type abnormal jumps are still not effectively suppressed by the transfer matrix. The Bootstrap verification shows that the reductions in illegal transition rates are consistently maintained for mistranslated segments, omitted segments, terminology inconsistency segments, cross-type abnormal jumps, and the overall transition rate, indicating stable structural differences between the proposed model and the comparison models throughout repeated resampling. Driven by the global constraints of CRF and the temporal features of BiLSTM, the model in this paper maintains the lowest illegal transition rate among all compared models, and the Bootstrap verification supports the stability of this advantage across repeated resampling. The boundary integrity and type consistency of the BIO structure remain stable throughout the evaluation process, providing consistent support for translation error localization in cultural heritage terminology.

### 4.5 Interpretability of cross-linguistic attention

**Figure 7:**
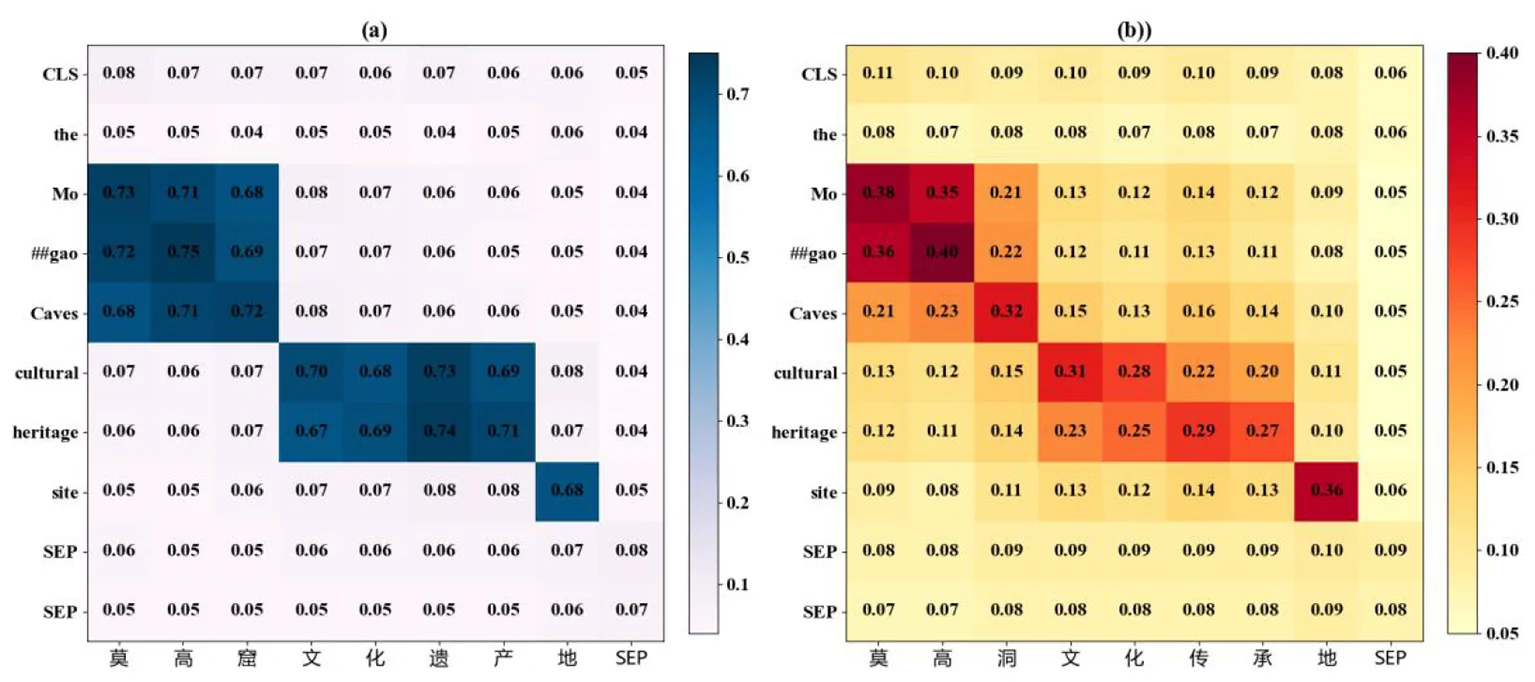
Heatmap of cross-linguistic semantic attention distribution. **(a)** Attention distribution under correct semantic mapping. **(b)** Attention distribution under incorrect semantic offset.

The quantitative evaluation of performance metrics verifies the model's detection effectiveness. However, whether the model truly forms a cross-linguistic semantic alignment discrimination mechanism for cultural heritage terms still needs to be verified through visualization analysis of attention distribution. To this end, the attention matrix output from the last Transformer layer of the BERT model is extracted. For two typical samples—correct semantic mapping and incorrect semantic offset—the attention weight distribution between the positions of English source terms and Chinese translation characters is statistically analyzed. All attention results are normalized and mapped to a unified color space to construct a cross-linguistic semantic attention heatmap. The comparison of attention distribution in correct and incorrect translation scenarios is shown in Figure 7.

Figure 7 illustrates the differences in attention distribution between scenarios with correct semantic mapping and incorrect semantic shift. In Figure 7a, a highly concentrated attention band is formed between English cultural heritage terms and their corresponding Chinese translations, while the weights of non-term regions remain at a low level. A significant weight contrast exists between the two, indicating that the bilingual splicing input structure drives BERT to learn stable term-level cross-lingual correspondences within a unified semantic space, enabling the model to form a clear attention bias in cross-lingual anchoring of cultural heritage terms. In Figure 7b, the attention distribution shows a discretization trend, with a decrease in the concentration of weights in the term correspondence regions. The originally stable term alignment focus region shows significant weakening and dispersion, with weights forced to disperse to surrounding non-term characters to seek semantic compensation. This discretization pattern directly reflects the model's perception of cross-lingual semantic mismatch—when there is a semantic shift between the translated term and the original text, BERT cannot establish a stable term alignment relationship in the bilingual sequence, thus triggering subsequent incorrect label predictions for that position by BiLSTM and CRF. The systematic differences in attention distribution between the two types of scenarios indicate that the model in this paper has formed a cultural heritage terminology error discrimination mechanism based on cross-linguistic semantic alignment during the training process, and there is an inherently consistent structural logic between attention-focusing behavior and detection results.

To further examine the relationship between attention distribution and different translation errors, the average normalized attention weights assigned to aligned terminology positions are calculated across the test set. Mistranslation samples exhibit the largest decrease in attention concentration, followed by terminology inconsistency and omission. The reduction in attention concentration is consistently associated with incorrect semantic alignment, indicating that dispersed attention distribution corresponds to weakened bilingual semantic correspondence rather than random attention variation.

## 5 Conclusion

This paper addresses the issues of cultural semantic shift, omissions, and inconsistencies in translation of cultural heritage terminology between English and Chinese. It constructs an automatic translation error detection model that integrates BERT cross-lingual semantic encoding, BiLSTM long-distance temporal modeling, and CRF global structured decoding. Through bilingual concatenated input, character-level BIO sequence annotation, and domain-specific fine-tuning strategies, it achieves unified identification and boundary localization of cultural heritage terminology errors. Experimental results show that the proposed model achieves an F1 score of 92.8% in error fragment-level evaluation, an improvement of 11.5 percentage points compared to the BiLSTM-CRF model, indicating that cross-lingual pre-trained semantic representation and structured sequence modeling have a significant positive impact on cultural heritage terminology error detection. In different error types, the F1 scores for mistranslation, omission, and inconsistency are 93.12%, 91.47%, and 90.86%, respectively, reflecting the model's stable ability to identify complex cultural semantic shifts and textual terminology consistency issues. In the BIO tag structure legality analysis, the illegal tag transfer rate decreases to 0.03%, effectively suppressing the “O → I” class boundary breakage and cross-type abnormal jump problems, and enhancing the integrity and structural consistency of the error localization results. Cross-linguistic attention heatmaps further validate that the model has formed a stable English-Chinese terminology semantic alignment mechanism, enabling the quality detection of cultural heritage terminology translation to shift from traditional manual verification to structured intelligent recognition, providing reliable technical support for the international dissemination of cultural heritage, the construction of multilingual digital resources, and the quality control of professional translation.

The present study evaluates the proposed model only on English Chinese translation because the experimental corpus is constructed for this language pair. The applicability of the proposed framework to other language pairs has not been investigated in the current study. Since different language pairs exhibit distinct linguistic structures and translation characteristics, additional multilingual datasets and experimental validation are required to further examine the generalization performance of the proposed model.

## Data Availability

The original contributions presented in the study are included in the article/supplementary material, further inquiries can be directed to the corresponding author.
